# Investigating the effect of providing monetary incentives to participants on completion rates of referred co-respondents: An embedded randomized controlled trial

**DOI:** 10.1016/j.conctc.2024.101267

**Published:** 2024-02-08

**Authors:** Abby Dunn, James Alvarez, Amy Arbon, Stephen Bremner, Chloe Elsby-Pearson, Richard Emsley, Christopher Jones, Peter Lawrence, Kathryn J. Lester, Natalie Morson, Nicky Perry, Julia Simner, Abigail Thomson, Sam Cartwright-Hatton

**Affiliations:** a University of Sussex, Falmer, United Kingdom; b University Hospitals Sussex NHS Foundation Trust, Brighton, United Kingdom; c Brighton and Sussex Medical School, Brighton, United Kingdom; d King's College London, London, United Kingdom; e University of Southampton, Southampton, United Kingdom

**Keywords:** Incentives, Trial design, RCT, SWAT, Co-respondent

## Abstract

**Background:**

The use of a second informant (co-respondent) is a common method of identifying potential bias in outcome data (e.g., parent-report child outcomes). There is, however, limited evidence regarding methods of increasing response rates from co-respondents. The use of financial incentives is associated with higher levels of engagement and follow-up data collection in online surveys. This study investigated whether financial incentives paid to index participants in an online trial of a parenting-focused intervention, would lead to higher levels of co-respondent data collection.

**Methods:**

A study within a trial (SWAT) using a parallel group RCT design. Participants in the host study (an RCT of an online intervention) were randomised into one of two SWAT arms: received/did not receive a £15 voucher when referred co-respondent completed baseline measures. Primary outcome was completion (No/Yes) of Spence Children's Anxiety Scale (SCAS or SCAS-Pre) at baseline. Additional analysis explored impact of incentives on data quality.

**Results:**

Intention to treat analysis of 899 parents (183 co-respondents) in the no-incentive arm, and 911 parents (199 co-respondents) in incentive arm. Nomination of co-respondents was similar between incentive arms. The RR for the incentive arm compared to the no incentive arm was 1.13 (95% CI: 0.91 to 1.41, p = 0.264) indicating that incentives did not impact completion of outcomes by consented co-respondents. There were no indications of different data quality between arms.

**Discussion:**

The finding that payment of financial incentives to index participant does not lead to greater levels of co-respondent outcome completion suggests that careful consideration should be made before allocating resources in this way in future trials.

**Trial registration:**

The host study was registered at Study Record | ClinicalTrials.gov and the SWAT study was registered in the SWAT Store | The Northern Ireland Network for Trials Methodology Research (qub.ac.uk): SWAT number 143: Filetoupload,1099612,en.pdf (qub.ac.uk).

## Highlights

• Co-respondents are a common method of collecting data on child outcomes.
• There is little evidence on how to promote data collection from co-respondents.
• Payment is associated with increased referral and response rates in online surveys.
• We found that paying participants did not lead to increased data collection from co-respondents.
• Paying participants did not have an impact on data quality.

## 1 Introduction

Parent-report measures are a widely used method of gathering data on child outcomes. However, it is a form of data collection with high potential for bias: for example, parents who are themselves anxious report higher levels of fear in their children compared with observer reports [1]. One method of identifying potential bias in parent-report measures is to use multiple informants. Gathering a second set of ratings, for example from the child, a teacher, or another familiar adult, allows the data to be triangulated [[2], [3], [4]].

In our Parenting with Anxiety study (PWA https://www.researchprotocols.org/2022/11/e40707), we decided to seek child outcome data from second informants. In this study, parents with self-identified elevated levels of anxiety participated in a randomised controlled trial of a preventative online parenting intervention designed to reduce the likelihood of their children developing anxiety. The primary trial outcome was children's anxiety symptoms and, given the ages of the children (as young as two years), the index parent (will from this be point referred to as ‘parent’) was responsible for reporting on this. However, we were concerned that parents' high levels of anxiety might bias their responses to our child anxiety outcome measures. Hence, we invited parents to nominate an adult who was familiar with their child to complete an additional child anxiety questionnaire. However, the success of this approach was dependent on a) the index parent's willingness to identify and invite a co-respondent and b) the co-respondent's willingness to accept the invitation and complete the measures.

The use of financial incentives to facilitate recruitment and retention of *index* participants in research studies (i.e., direct recruitment of a participant, not via a third party) has been evaluated extensively: financial incentivisation can take various forms including voucher and cash payments and lotteries through which the incentives are randomly allocated to a proportion of participants (see Parkinson for overview of literature [5]. A 2014 Cochrane review of strategies to improve retention in randomised controlled trials (RCTs) identified 38 trials and found that both the offer and provision of financial incentives, compared with other methods (i.e. amendment to questionnaire design), was associated with more questionnaire completion including for electronic questionnaires [6]. A follow-up review in 2023 found the evidence on the effect of monetary incentives was inconclusive, with indications that payment increased retention compared with no incentive and that higher value incentives may be more effective. However, the authors highlighted that they had low confidence in the effect sizes for these findings due to issues around design and other variables [7]. A meta-analysis of the use of incentives to promote health survey responses generated similar findings, with financial incentives more likely to generate responses than other such as charity donation [8]. Looking specifically at online trials, in an RCT of an online parenting course for parents of young anxious children, the offer of being entered into a prize draw to receive a £30 voucher was associated with an 11% increase in follow-up data collection in both the intervention and control arms [9]. Investigating the effects of different values and delivery methods, Khadjesari and colleagues determined that offering to give participants a £10 voucher when they completed their 12-month follow-up questionnaires led to a 9% greater response rate compared with an un-incentivised control, but that offering a £5 voucher did not have an equivalent effect [10].

There is no research, to our knowledge, that has explored the recruitment and retention of co-respondents into randomised controlled trials. Evidence relating to methods that might improve recruitment and retention of co-respondents in other designs is sparse. In a study on financial incentives for snowball sampling for a large online questionnaire, which involved an index participant sending on an invitation to complete the survey to members of their online social network, a fixed incentive of $0.17 was associated with a 100-times greater number of surveys shared compared with a higher financial value lottery incentive (1% chance of winning $17) [11]. However, when index participants selected a reward for their own survey completion, the lottery was substantially more popular. Within the digital marketing domain, referral is a common method to attain customers. In a large field experiment conducted on customers of an online shopping platform, which provided cashback on purchases, higher value financial incentives led to higher levels of new customer referrals, new member sign-ups and new buyers. Furthermore, the referral rate was higher when the referring individual was aware that their remuneration was higher than the one offered to the recipient [12].

The current study was designed in response to the lack of literature on maximising co-respondent data collection in RCTs. The literature on trial retention and questionnaire completion indicated that financial incentives were a plausible method to increase referrals and co-respondent responses. We used an embedded Study Within a Trial (SWAT) design to investigate the effect of incentivising index participants on data collection from a second informant. At the start of the trial, all index participants were invited to nominate someone to provide data on their child. A randomised half of these index participants did so with the expectation that they would receive a £15 voucher should their co-respondent complete our measures. We anticipated that the incentive arm, compared to the control arm, would nominate more co-respondents because incentives are associated with greater levels of participant engagement and because incentive arm participants might also be more inclined to ‘*nudge’* the co-respondent to complete measures (given that their own remuneration was contingent upon the co-respondent completing these). We also planned to measure whether and differences between arm were maintained at six-month follow-up and to evaluate whether the payment of incentives had any impact on data quality.

Given the nested nature of the study within a trial (SWAT) design, and the need to ensure that the SWAT did not negatively impact data collection for the main study, we offered all co-respondents a £10 voucher on completion of their measures at each time point (this was in addition to the payment to the person who referred them). Given that the evidence suggests more nominations when the nominator is paid more than the nominee, we decided that the payments to the nominator (£15) should be more than to the nominated person (£10).

We hypothesised that payments to index participants would lead to the following, compared to the control arm:

● higher rates of completion of co-respondent baseline measures in the incentive arm.
● higher rates of completion of co-respondent six-month follow-up measures in the incentive arm.
● higher rates of nomination of a co-respondent in the incentive arm.
● higher rates of consented co-respondents in the incentive arm.

We also planned an exploratory investigation into whether payment had an effect on the quality of data returned by co-respondents.

## 2 Methods

### 2.1 Aim and design

This study within a trial (SWAT) used an embedded parallel group RCT design to investigate the impact of paying host trial index participants on the nomination and subsequent engagement of co-respondents. The SWAT was embedded within an RCT of an online intervention designed to limit the impact of parental anxiety on child outcomes [13]. Host trial participants (parents) were asked to nominate a co-respondent who would themselves participate in the study by completing a set of measures on child anxiety. This paper is reported in accordance with guidelines for reporting embedded recruitment trials based on the Consolidated Standards for Reporting Trials (CONSORT) statement 2010 (for CONSORT checklist see supplementary material) [14].

### 2.2 Participants

A sample size of 1754 participants was calculated to provide 90% power for the main objective in the host trial. All participants in the host trial were included in the SWAT. Eligibility criteria for the host trial participants were that they were anxious adults (aged 16 and above) who had children aged 2–11 years. Full host trial recruitment procedures can be found in the trial protocol (https://www.sciencedirect.com/science/article/pii/S2451865423000364).

In the SWAT, index participants (parents) were randomised 1:1 to payment and non-payment arms. The sample size for this SWAT was expected to equal the sample size that was calculated to provide adequate power for the key objective of the host of the trial (N = 1754). The expectation was for a co-respondent baseline questionnaire response rate of 65% (n = 570) where the incentive was offered and 55% (n = 482) in the non-incentivised arm. With 1754 participants, we would have >95% power to detect this 10% difference between arms. Eligibility criteria for co-respondents were that they were aged over 16 and knew the child well enough to answer a brief questionnaire about the child's feelings and behaviours. Index participants were advised that co-respondents could be family members, friends, or any other relationship, but, for ethical reasons, could not be individuals with whom the participant had a monetised relationship (e.g., babysitter).

### 2.3 Interventions

The host study was a community recruited online study for which all study activities took place on a secure online platform. Participants self-referred into the host study and all participants were given the option to nominate a co-respondent. All host study index participants were randomised into one of two SWAT arms (1:1 ratio) where they either received or did not receive a £15 voucher when a referred co-respondent completed the baseline assessment measures. This randomisation was done without their knowledge. All co-respondents received a £10 voucher on completion of measures at baseline and six-month follow-up. Participant activities took place as follows:

i. Index participant received summary information.
ii. Index participant screened against inclusion/exclusion criteria.
iii. Those meeting inclusion criteria received detailed information about the host study and gave consent online.
iv. Index participant randomised into one of two SWAT arms: Incentive or No Incentive.
v. Index participants asked to provide details of a co-respondent who was then contacted by email. An index participant could also choose not to refer or to make a referral later.
vi. Index participant completed baseline measures.
vii. Index participant randomised into Intervention/Control arm of main trial (Parenting with Anxiety).
viii. Forty-eight hours after index participant had completed measures, the nominated co-respondent was emailed information about the host study and, if willing, gave consent online.
ix. Co-respondent completed baseline measures.
x. Host participant paid, if they were in the Incentive arm of SWAT.
xi. Six months post consent, host participant and co-respondent invited to complete follow-up measures.

### 2.4 Outcomes

Outcome measures were administered online at baseline (T1) and 6-months post consent (T2). For host trial participants, the measures specified below were part of a larger battery of assessments (these are listed in full in the main trial protocol [13]). The measures completed by co-respondents were determined by their relationship to the index child: All co-respondents completed the Spence Children's Anxiety Scale (SCAS or Preschool SCAS (SCAS-Pre) according to the child's age) [15,16]. These parallel instruments are used widely in clinical research as an assessment of child anxiety symptoms, are acceptable to parents and have good reliability and validity. Only co-respondents with parental responsibility (co-parents) also completed the SCARED-A, a 71-item assessment of adult anxiety symptoms, which is strongly correlated with the ADIS-IV-L diagnostic interview schedule, and the CPBQ, which measures anxiogenic parenting behaviours [[17], [18], [19]]. Co-respondents without parental responsibility completed the Generalised Anxiety Disorder Assessment (GAD-7) a seven-item screening measure for anxiety disorder which was administered instead of the SCARED-A to reduce response burden [20].

#### 2.4.1 Primary outcome

The primary outcome for the SWAT was completion (No/Yes) of the Spence Children's Anxiety Scale (SCAS or SCAS-Pre), the primary outcome measure in the host trial. For the purposes of analysis, completion was defined as being able to calculate a score for SCAS or SCAS-Pre where calculation was contingent on being able to calculate a score for all subscales (each required >80% items).

#### 2.4.2 Secondary outcomes

Secondary SWAT outcomes were:

● co-respondent nomination by host trial participants (as measured by host trial participant provision of co-respondent email address)
● co-respondent consent.
● data quality
  o concordance between measures (intraclass correlations and bias/agreement between host-trial participant and co-respondent)
  o time taken to complete the SCAS/SCAS-Pre.

### 2.5 Randomisation

Block randomisation, in blocs of 4, 8, 12, 16 and 20, occurred simultaneously with (but independently of) randomisation in the host study, so that host trial participants were allocated to one of four groups (Host Intervention arm and SWAT Incentive arm; Host Intervention arm and SWAT No Incentive arm; Host Control arm and SWAT Incentive arm; Host Control arm and SWAT No Incentive arm). Host trial participants were aware of their SWAT condition (i.e., whether they would be paid for nomination or not) prior to nominating a co-respondent but were unaware there was an alternative condition. Participants were made aware of this passive deception in a debrief letter issued once data collection had finished.

### 2.6 Approvals

Ethical approval has been obtained for both the host study, and this SWAT from the Sponsor's Cross Schools Ethics Committee (C-REC). The host study was registered at Study Record | ClinicalTrials.gov and the SWAT study was registered in the SWAT Store | The Northern Ireland Network for Trials Methodology Research (qub.ac.uk): SWAT number 143: Filetoupload,1099612,en.pdf (qub.ac.uk).

### 2.7 Statistical methods

**Fig. 1:**
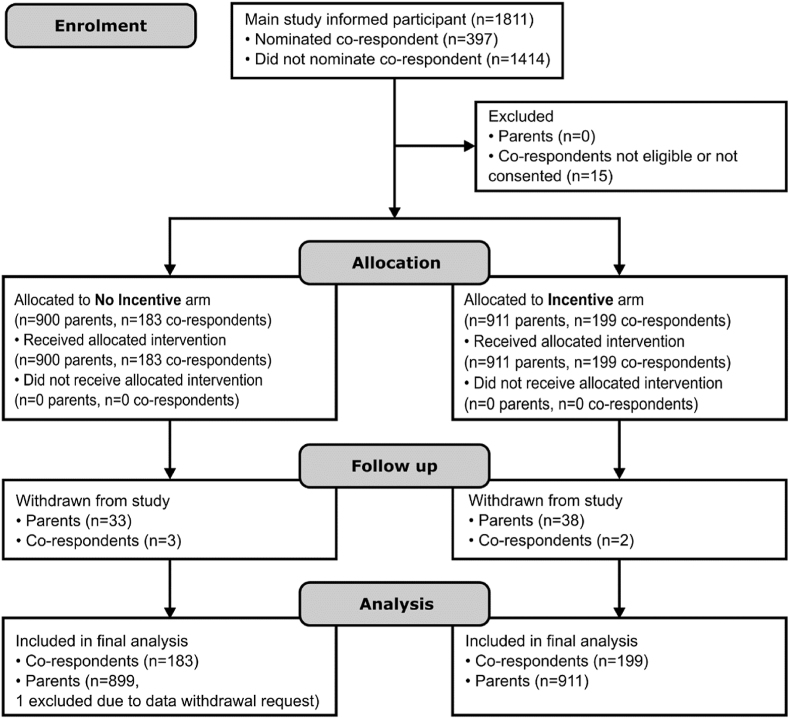
SWAT CONSORT flow diagram.

Analyses were conducted in Stata 17.0 following intention to treat (ITT) principles [21]. We calculated standardised z-scores to allow for variation in item number, response scales and scoring between the SCAS and SCAS-Pre. For purposes of analysis, we defined completion as non-missing data for at least 80% of the primary outcome (SCAS or SCAS-Pre.), with “Prefer not to answer” responses set as missing. Nominated, consented and completed co-respondents are summarised at baseline and six-month follow-up in Fig. 1.

We planned to model completion of co-respondent outcomes at baseline and 6-months using multivariable log-binomial regression models fitted for the primary outcome (completion) with a random effect for participant, and SWAT trial arm and time point (baseline/m6) as fixed effects. However, these models failed to converge. Instead, we fitted a Poisson model with robust standard errors to estimate the risk ratio and estimate its 95% CI [22]. We used a mixed effects logistic regression model to estimate the odds ratio, which is reported with 95% confidence intervals.

Data from co-respondents was modelled using log-binomial and logistic regression models. We assessed data quality with intraclass correlations calculated for parent and co-parent outcomes and using Bland-Altman plots which summarised and graphically displayed agreement between parent and co-respondents. We also calculated median time taken per question on the SCAS and SCAS-Pre scales and compared them by incentive arm. An interim data quality assessment was carried out once 170 participants had been randomised and then repeated as part of the final analysis.

## 3 Results

### 3.1 Interim analysis

We conducted an interim analysis to assess whether host study data quality was being compromised by inclusion of the SWAT, and the SWAT would have been terminated if this was found to be the case. We investigated the data quality after 170 co-respondents (co-parents = 126; other co-respondents = 44) had been randomised (No incentive group = 89; Incentive group = 81). The analysis found adequate data quality across SWAT arms - similar levels of data completion between the SWAT trial arms (incentive/no-incentive) and no obvious differences in questionnaire completion times or variability of outcomes scores. Bland-Altman plots showed bias on the SCAS-P/Preschool-SCAS with co-respondents scoring higher (more child anxiety symptoms) than host trial participants however the amount of bias was similar in the two SWAT trial arms. Therefore, the SWAT was continued.

### 3.2 Main analysis

#### 3.2.1 Participants

Recruitment into the SWAT ran from February 2021 to September 2022 at which point the host trial recruitment target had been met. Follow-up data was required from parents and co-respondents six-months post consent. Of 1811 host trial participants (parents), 900 were allocated to the no-incentive arm and 911 to the incentive arm. In total, 397 index parents nominated a co-respondent. Fifteen nominated co-respondents were ineligible or did not consent. One index parent withdrew their data from analysis, with the result that 899 parents and 183 co-respondents in the no-incentive arm, and 911 parents and 199 co-respondents in the incentive arm were subject to the final ITT analysis. The full participant flow is described in Fig. 1.

Most co-respondents were co-parents to the index child (Overall: n = 288 (75.4%); No incentive: n = 134 (73.2%); Incentive: n = 154 (77.4%). Baseline demographics and clinical characteristics can be found in Table 1.

**Table 1:** Demographic and clinical characteristics of co-respondents.

	No incentive arm (n = 183)			Incentive arm (n = 199)			Overall (n = 382)		
	Median	IQR	n	Median	IQR	n	Median	IQR	n
**Age**	41.0	37.0 to 49.0	183	40.0	36.0 to 47.0	196	41.0	36.0 to 47.0	379
	**n**	**%**		**n**	**%**		**n**	**%**	
**Gender**									
Male	122	67.4		139	70.6		261	69.0	
Female	59	32.6		56	28.4		115	30.4	
I prefer not to say	0	0.0		2	1.0		2	0.5	
Total	181	100.0		197	100.0		378	100.0	
**Ethnicity**									
English/Welsh/Scottish/Northern Irish/British	157	86.7		165	84.2		322	85.4	
Irish	2	1.1		0	0.0		2	0.5	
Any other White background	14	7.7		16	8.2		30	8.0	
White and Black Caribbean	1	0.6		1	0.5		2	0.5	
White and Black African	0	0.0		1	0.5		1	0.3	
White and Asian	1	0.6		1	0.5		2	0.5	
Any other Mixed/Multiple ethnic background	2	1.1		2	1.0		4	1.1	
Indian	1	0.6		1	0.5		2	0.5	
Chinese	0	0.0		2	1.0		2	0.5	
Any other Asian background	0	0.0		1	0.5		1	0.3	
African	1	0.6		0	0.0		1	0.3	
Caribbean	0	0.0		1	0.5		1	0.3	
Any other Black/African/Caribbean background	1	0.6		2	1.0		3	0.8	
Arab	1	0.6		1	0.5		2	0.5	
Any other ethnic group	0	0.0		2	1.0		2	0.5	
Total	181	100.0		196	100.0		377	100.0	
**Financial status**									
Comfortable	81	44.8		104	53.1		185	49.1	
Managing	90	49.7		78	39.8		168	44.6	
Struggling	10	5.5		14	7.1		24	6.4	
Total	181	100.0		196	100.0		377	100.0	
**Education**									
Left school before 16	5	2.8		4	2.0		9	2.4	
Left school at 16	13	7.2		20	10.2		33	8.8	
Left school 17/18	16	8.8		18	9.2		34	9.0	
Completed college	28	15.5		40	20.4		68	18.0	
Completed university	119	65.7		114	58.2		233	61.8	
Total	181	100.0		196	100.0		377	100.0	
**Co-respondent relationship to child**									
Parent	134	73.2		154	77.4		288	75.4	
Grandparent	31	16.9		20	10.1		51	13.4	
Other relation	7	3.8		15	7.5		22	5.8	
Friend	6	3.3		4	2.0		10	2.6	
Other	5	2.7		6	3.0		11	2.9	
Total	183	100.0		199	100.0		382	100.0	

#### 3.2.2 Primary outcome: Co-respondent completion of Spence Children's Anxiety Scale (SCAS/SCAS-P)

The models for the primary outcome were fitted for 1810 consented participants of the main study, 382 of whom had consented co-respondents able to complete measures at baseline and six-month follow-up, In the no incentive arm 169/899 (18.8%) completed outcomes at baseline compared to 194/911 (21.3%) in the incentive arm. At six-month follow-up 148/899 (16.5%) in the no incentive arm completed outcomes, compared to 163/911 (17.9%) in the incentive arm. The RR for the incentive arm compared to the no incentive arm was 1.13 (95% CI: 0.91 to 1.41, p = 0.264) and the OR was 1.90 (95% CI: 0.83 to 4.34, p = 0.127), indicating that incentives did not impact completion of outcomes by consented co-respondents.

Completion of measures within arms was slightly higher at T1 than T2, with the trend similar across arms (as percentage of parents in study, no incentive: 19.6% (176/899) at T1 and 16.8% (151/899) at T2, incentive: 21.5% (196/911) at T1 and 17.9% (163/911) at T2; as percentage of co-respondents consented, no incentive: 92.3% (169/183) at T1 and 80.9% (148/183) at T2, incentive: 97.5% (194/199) at T1 and 81.9% (163/199) at T2).

#### 3.2.3 Secondary outcomes

##### 3.2.3.1 Co-respondent nomination and consent

Nomination of co-respondents was similar between incentive arms (no incentive: 21.0% (189/899), incentive: 22.8% (208/911)). The RR for the incentive arm compared to the no incentive arm was 1.09 (95% CI: 0.91 to 1.29, p = 0.353) and the OR was 1.11 (95% CI: 0.89 to 1.39, p = 0.353), indicating that incentives did not impact nomination of co-respondents by index parents.

Consent of co-respondents was also similar in both arms (as percentage of parents in study, no incentive: 20.4% (183/899), incentive: 21.8% (199/911); as percentage of co-respondents nominated, no incentive: 96.8% (183/189), incentive: 95.7% (199/208)). The RR for the incentive arm compared to the no incentive arm was 1.07 (95% CI: 0.90 to 1.28, p = 0.438) and the OR was 1.09 (95% CI: 0.87 to 1.37, p = 0.438), indicating that incentives did not impact the consent of co-respondents.

### 3.3 Data quality

There were no indications of different data quality between arms, based on agreement and bias summarised by ICCs and Bland-Altman plots. Time taken by all co-respondents per question on SCAS/SCAS-P was similar between arms at each time point, and similar overall across time points. Tables and figures reporting data quality analysis are presented in supplementary materials.

### 3.4 Harms

No harms were recorded in either trial arm nor in the host trial.

## 4 Discussion

The current study was designed to examine whether paying randomised controlled trial participants, compared to not paying them, would increase the likelihood of them nominating a secondary co-respondent, and of that co-respondent providing data. The results indicate that paying host trial participants has no detectable effect on the nomination, consent or data completion of co-respondents. However, the study demonstrated that incentivising trial participants in this manner, had no impact (beneficial *or* detrimental) on the quality of data provided by nominated co-respondents. Given the paucity of research in this area of trial design, these findings provide a valuable signal which can be developed in future research with the potential to inform incentive allocation in randomised controlled trials (RCTs).

Given the literature which indicates that financial incentives are the most effective way of promoting data completion and retention in research studies, it is surprising that payment did not result in elevated co-respondent referral or data completion [6,8]. The lack of difference between arms may be explained by the lack of control referring participants had over the outcome (co-respondent data completion) upon which their own payment was contingent. While research into incentivised referral schemes outside randomised controlled trials designs suggests that incentives are associated with increased referral rates, these studies largely used designs where participants could make multiple referrals, and where payment was made when the referral was made (e.g. Refs. [12,23]). Within the current study, referees could refer only one co-respondent. Furthermore, payment was contingent on two factors, one fully within their own control, referring the co-respondent *and* one outside their control, the co-respondent completing measures. This ‘both/and’ requirement may have limited the motivational impact of the incentive on the index participant. Drawing upon the behavioural economics literature around mechanisms of action (MA), the process through which a behaviour occurs, this uncertainty may have been associated with reduced belief about the likelihood of consequences of an occurrence. In doing so, it suppressed the willingness of a participant to take the requested action (See Schenk 2023 for ontology of MoAs [24]). Given extant findings that a minimum monetary threshold must be met for incentives to affect co-respondent completion rates, it is also possible that the incentive offered within this trial was insufficient [6,25]. A larger incentive might have galvanised more index participants to refer and potentially to encourage them to remind co-respondents to complete data.

As outlined above, there is negligible research into referral incentives within clinical mental health trials. The literature focused on referrals is largely focused on referral into activities for which the participant has limited personal investment (e.g. online surveys and shopping) [12]. In contrast, participants in the Parenting with Anxiety study were involved in research related to their own mental health difficulties and their children's mental health and were requesting a co-respondent to answer questions about their child's mental health. How the participant felt the referral scheme would reflect on them to people they invited, was the biggest factor predicting the success of a referral reward programmes within the banking sector. In the cross-sectional study which integrated routinely recorded data banking with supplementary attitudinal questionnaires, this metaperception along with perceived attractiveness of the reward fully mediated the effects of incentives on referral likelihood [23]. In the case of the current study, the association with mental health may have had a suppressive effect on the index participants willingness to refer a co-respondent. Furthermore, the highly personal nature of this participation may have limited the impact of the incentive – if a participant felt willing and motivated to invite a second respondent they would do so with or without the offer of payment. Models of motivation which incorporate the interplay of intrinsic and extrinsic motivation indicate that co-respondent referral within this context is associated with activation of the intrinsic motivation system, in particular the ‘purpose’ component through which motivation is attached to performing an action which has wider societal benefits (e.g., Refs. [26,27]. Financial incentives, which seek to engage extrinsic motivation, have limited impact where individuals already have high levels of intrinsic motivation to perform a task. Indeed, in some cases payment can reduce intrinsic motivation and overall task performance [28,29]. Thus, it is possible that the similarity between co-respondent referral rates across the two arms of the current study reflects the proportion of individuals within each arm who had high levels of intrinsic motivation to carry out the task. Research on the use of incentives in population surveys found that actions targeting intrinsic motivation (e.g., redesigning the questionnaire to stress the voluntary nature of participation) are most effective in improving response rates, however these can be difficult to engineer [30]Furthermore, while intrinsic motivation can be increased through extrinsic rewards, these must be offered directly after the task is completed, a condition not met within the current study [31].

The current study does not offer evidence that financial incentives offered to a participant increase co-respondent data collection. This runs counter to our hypotheses and the wider literature and should be accounted for in the design of future mental health RCTs, where operating under assumptions about the effectiveness of incentives may lead to poor allocation of resources. Research that uses larger monetary incentives, and/or that rewards on the point of referral rather than after co-respondent data completion, would be useful extensions of the work and of potential benefit in future study design. Alongside this, investigation into participant motivation in nominating a co-respondent would enable future trial designs to more effectively engage with and activate the relevant motivational system.

### 4.1 Strengths and limitations

Using a nested study within a trial (SWAT) was an effective method of delivering a large study with minimal cost or respondent burden. The aim of the SWAT was a further understanding of the effect of participant payment on the capture of second informant data within an RCT. With a large sample size, it effectively demonstrated that a small payment is an ineffective way of obtaining more co-respondent data. However, it also demonstrated that such payment has no impact on the quality of data returned by co-respondents. As discussed, this has the potential to have practical utility for future researchers seeking to incorporate second informant data. However, given the importance of data collection in RCTs, we would advise caution until these findings have been replicated in subsequent research. There would also be considerable value in identifying the possible interaction between incentivisation and other features which may impact on data collection and retention such as trial design and follow-up duration (see Gaunt 2023 for review [32]).

While the study is both novel and useful there are some limitations that could be addressed in future research. The host trial ‘Parenting with Anxiety’ was an online study for which the participants were parents who self-identified as high in anxiety. These features may limit the generalisability of the findings in particular with regard to the potential impact of the mental health of the participants on their willingness to refer. It is also possible that there may be a differential impact of payment on referral activities in face-to-face clinical mental health trials.

The sample for the Parenting with Anxiety SWAT study was large as clinical RCTs go. It was, however, considerably less than was anticipated in the original SWAT design. Fewer participants in the host trial referred a co-respondent than was expected and it would be of clear benefit to understand why referral rates were lower than predicted.

## Funding sources

This work was supported by 10.13039/100001201Kavli Trust (grant 38/19).

## CRediT authorship contribution statement

**Abby Dunn:** Investigation, Methodology, Project administration, Supervision, Validation, Writing – original draft, Writing – review & editing. **James Alvarez:** Data curation, Software, Writing – review & editing. **Amy Arbon:** Project administration, Supervision, Writing – review & editing. **Stephen Bremner:** Data curation, Formal analysis, Visualization. **Chloe Elsby-Pearson:** Conceptualization, Investigation, Writing – review & editing. **Richard Emsley:** Conceptualization, Formal analysis, Methodology. **Christopher Jones:** Formal analysis, Visualization, Writing – review & editing. **Peter Lawrence:** Conceptualization, Methodology, Writing – review & editing. **Kathryn J. Lester:** Conceptualization, Investigation, Methodology, Writing – review & editing. **Natalie Morson:** Conceptualization, Writing – review & editing. **Julia Simner:** Conceptualization, Methodology, Writing – review & editing. **Abigail Thomson:** Investigation, Writing – review & editing. **Sam Cartwright-Hatton:** Conceptualization, Funding acquisition, Investigation, Methodology, Project administration, Supervision, Writing – review & editing.

## Declaration of competing interest

The authors declare the following financial interests/personal relationships which may be considered as potential competing interests.

SC-H designed the digital intervention and funded its development.

## Data Availability

Data will be shared on the Figshare data repository once the main trial paper has been published
