# Supplementary material for: Investigating the effect of providing monetary incentives to participants on completion rates of referred co-respondents: An embedded randomized controlled trial
Source: Contemp Clin Trials Commun. 2024 Feb 8;38:101267. doi: 10.1016/j.conctc.2024.101267 (PMC10899055; doi:10.1016/j.conctc.2024.101267)
Supplement: Multimedia component 1 [file mmc1.docx]

Appendix A: Data quality evaluation

**Table 1:** ICCs for parent and co-parent outcomes (SCAS and CPBQ) by arm.

|  | **No Incentive** | | | **Incentive** | | | **Overall** | | |
| --- | --- | --- | --- | --- | --- | --- | --- | --- | --- |
| **Outcome** | **ICC** | **95% CI** | **n** | **ICC** | **95% CI** | **n** | **ICC** | **95% CI** | **n** |
| **SCAS** |  |  |  |  |  |  |  |  |  |
| Standardised overall score T1 | 0.590 | 0.488 to 0.693 | 152 | 0.613 | 0.523 to 0.704 | 178 | 0.602 | 0.534 to 0.670 | 330 |
| Standardised overall score T2 | 0.519 | 0.391 to 0.647 | 120 | 0.591 | 0.484 to 0.698 | 134 | 0.556 | 0.473 to 0.639 | 254 |
| **CPBQ** |  |  |  |  |  |  |  |  |  |
| Challenging behaviour T1 | 0.061 | -0.252 to 0.130 | 128 | 0.221 | 0.064 to 0.378 | 148 | 0.085 | -0.038 to 0.209 | 276 |
| Challenging behaviour T2 | 0.044 | -0.176 to 0.264 | 97 | 0.001 | -0.211 to 0.209 | 110 | 0.023 | -0.128 to 0.175 | 207 |
| Overinvolvement T1 | 0.195 | 0.024 to 0.367 | 129 | 0.262 | 0.110 to 0.414 | 149 | 0.234 | 0.120 to 0.348 | 278 |
| Overinvolvement T2 | 0.245 | 0.052 to 0.439 | 97 | 0.151 | -0.043 to 0.345 | 110 | 0.195 | 0.058 to 0.333 | 207 |
| Warmth T1 | 0.183 | 0.010 to 0.356 | 129 | 0.204 | 0.046 to 0.362 | 149 | 0.195 | 0.078 to 0.311 | 278 |
| Warmth T2 | 0.293 | 0.108 to 0.479 | 97 | 0.136 | -0.060 to 0.332 | 110 | 0.206 | 0.069 to 0.342 | 207 |
| Negativity T1 | 0.134 | -0.043 to 0.312 | 129 | 0.235 | 0.080 to 0.390 | 149 | 0.195 | 0.079 to 0.312 | 278 |
| Negativity T2 | 0.100 | -0.113 to 0.314 | 97 | 0.241 | 0.059 to 0.423 | 110 | 0.180 | 0.041 to 0.319 | 207 |
| Negative discipline T1 | 0.234 | 0.067 to 0.402 | 129 | 0.271 | 0.120 to 0.422 | 149 | 0.254 | 0.142 to 0.366 | 278 |
| Negative discipline T2 | 0.244 | 0.050 to 0.438 | 97 | 0.356 | 0.192 to 0.520 | 110 | 0.307 | 0.181 to 0.432 | 207 |
| Positive discipline T1 | 0.202 | 0.031 to 0.373 | 129 | 0.232 | 0.076 to 0.387 | 148 | 0.221 | 0.106 to 0.336 | 277 |
| Positive discipline T2 | 0.313 | 0.131 to 0.496 | 97 | 0.168 | -0.024 to 0.360 | 110 | 0.237 | 0.104 to 0.371 | 207 |

**Table 2**. Summaries of time taken per question on SCAS/SCAS-Pre, SCAS only and SCAS-Pre only at each time point, by arm.

|  | **No incentive arm** | | | **Incentive arm** | | | **Overall** | | |
| --- | --- | --- | --- | --- | --- | --- | --- | --- | --- |
|  | **Median** | **IQR** | **n** | **Median** | **IQR** | **n** | **Median** | **IQR** | **n** |
| **Time taken (seconds) per question at T1 on** |  |  |  |  |  |  |  |  |  |
| **SCAS or SCAS-Pre** | **6.8** | **5.1 to 10.4** | **169** | **6.7** | **5.1 to 9.8** | **193** | **6.7** | **5.1 to 10.0** | **362** |
| SCAS only | 6.6 | 5.1 to 10.2 | 83 | 6.5 | 5.1 to 8.7 | 103 | 6.6 | 5.1 to 9.5 | 186 |
| SCAS-Pre only | 6.9 | 5.1 to 11.5 | 86 | 7.3 | 5.3 to 10.2 | 90 | 7.1 | 5.3 to 10.6 | 176 |
|  |  |  |  |  |  |  |  |  |  |
| **Time taken (seconds) per question at T2 on** |  |  |  |  |  |  |  |  |  |
| **SCAS or SCAS-Pre** | **7.5** | **5.1 to 9.9** | **148** | **6.5** | **4.6 to 9.6** | **158** | **7.1** | **4.9 to 9.7** | **306** |
| SCAS only | 6.1 | 4.5 to 8.5 | 81 | 5.1 | 3.9 to 7.9 | 91 | 5.6 | 4.3 to 8.0 | 172 |
| SCAS-Pre only | 8.6 | 7.1 to 10.9 | 67 | 8.4 | 6.3 to 12.8 | 67 | 8.4 | 6.6 to 11.5 | 134 |
|  |  |  |  |  |  |  |  |  |  |
